# In-House Validated Map of Lymph Node Stations in a Prospective Cohort of Colorectal Cancer: A Tool for a Better Preoperative Staging

**DOI:** 10.1155/2022/1788004

**Published:** 2022-03-19

**Authors:** Patricia Simu, Ioan Jung, Laura Banias, Zsolt Zoltan Fulop, Tivadar Bara, Iunius Simu, Sebastian Andone, Raluca Ioana Stefan-van Staden, Catalin Bogdan Satala, Ioana Halmaciu, Simona Gurzu

**Affiliations:** ^1^Department of Radiology and Imaging, Clinical County Emergency Hospital, Targu Mures, Romania; ^2^Department of Pathology, George Emil Palade University of Medicine, Pharmacy, Sciences and Technology, Targu Mures, Romania; ^3^Department of Surgery, George Emil Palade University of Medicine, Pharmacy, Science and Technology, Targu Mures, Romania; ^4^Department of Neurology, George Emil Palade University of Medicine, Pharmacy, Sciences and Technology, Targu Mures, Romania; ^5^Laboratory of Electrochemistry and PATLAB, National Institute of Research for Electrochemistry and Condensed Matter, Bucharest, Romania; ^6^Department of Anatomy, George Emil Palade University of Medicine, Pharmacy, Sciences and Technology, Targu Mures, Romania; ^7^Research Center of Oncopathology and Transdisciplinary Research, George Emil Palade University of Medicine, Pharmacy, Sciences and Technology, Targu Mures, Romania

## Abstract

Preoperative staging of colorectal cancer (CRC) based on imaging techniques such as computed tomography (CT) or magnetic resonance imaging (MRI) is crucial for identification and then removal of the positive lymph nodes (LNs). The aim of this study was to evaluate the correlation between preoperatively seen morphologic criteria (number, size, shape, structure, borders, or enhancement patterns) and histopathological features of LNs using an in-house validated map of nodal stations. A total of 112 patients with CRC that underwent surgery were preoperatively evaluated by CT scans. The locoregional, intermediate, and central LNs were CT-mapped and then removed during open laparotomy and examined under microscope. The analysis of correlations was interpreted using the suspicious-to-positive ratio (SPR) parameter. The greatest correlation was found in tumors located in the sigmoid colon, descending colon and middle rectum; SPR value was 1.12, 1.18, and 1.26, respectively. SPR proved to be 0.59 for cases of the transverse colon. Regarding the enhancement type, the dotted pattern was mostly correlated with metastatic LNs (OR: 7.84; p < 0.0001), while the homogenous pattern proved a reliable indicator of nonmetastatic LNs (OR: 1.99; p < 0.05). A total of 1809 LNs were harvested, with a median value of 15 ± 1.34 LNs/case. Transdisciplinary approach of CRC focused on pre-, intra-, and postoperatively mapping of LNs might increase the accuracy of detecting metastasized nodes for tumors of the distal colon and middle rectum but not for those of the transverse colon. In addition to morphologic criteria, the enhancement pattern of LNs can be used as a predictor of nodal involvement improving the CT-based preoperative staging.

## 1. Introduction

Despite individualized therapy, colorectal cancer (CRC) remains one of the most prevalent digestive malignancies worldwide and the second leading cause of cancer-related deaths, with an increasing incidence in the last years [1–3]. Long overall survival rate (OS) is reported in patients with CRC diagnosed in early stages [2–4]. Screening programs are developed in most of the countries but lack of optimization of policy of screening and surveillance by colonoscopy lead to diagnosis of most of the cases in advanced stages [5, 6].

Although several modern prognostic parameters were proposed, the number of harvested lymph nodes (LN) same as the number of metastatic nodes (N status) and the rate of positive vs. removed nodes (lymph node ratio—LNR) remain the most important independent prognostic parameters [7–9]. The 5-year survival rate of 75-95% was reported for patients with CRC diagnosed in stages I or II (N0) compared to 30-68% in stages III or IV [7, 8, 10]. Furthermore, 20-30% of the N0-staged recurrent cases with completely excised tumors and free resection margins (R0) could be linked to occult LN metastases [11]. An accurate imaging evaluation of the LN status is crucial for choosing extensive lymphadenectomy, pre- or postsurgery chemo- and/or radiotherapy, as well as neoadjuvant therapy.

Although LN status and identification of synchronous CRCs can be successfully done preoperatively using imaging methods such as computed tomography (CT) and magnetic resonance imaging (MRI) [9], the reported sensitivity and specificity for LN mapping via CT-scan is about 71% and 41%, respectively [12]. Several criteria have been proposed for a more accurate evaluation of LN status. LN size has been used as a predictor for positive LNs, with a threshold size of 10 mm, but the sensitivity and specificity do not exceed 71% and 67%, respectively [13–15]. In other studies, it was shown that most metastatic LNs were <5 mm whereas those beyond 10 mm were enlarged due to a good host inflammatory response [10, 11].

Japanese Society for Cancer of the Colon and Rectum, respectively, Yamamoto et al., proposed to use, as a prognostic parameter, a map of LN stations which was designed based on the localization of the LNs. They divided the LN stations into three main categories: locoregional, stations near great vessels, and stations located at the origin of great vessels [16]. Metastases in the stations located at the origin of great vessels upgrade staging at stage IV [17].

For contrasting CT-scan identification of the LN stations and suspected nodes, size, roundness, heterogeneity, and contour irregularity should be checked [18]. Based on combined features, Miao et al. proposed six patterns of internal enhancement: homogenous, striped, spotted, core, rim, and heterogenous [12]. Heterogeneity and rim pattern may correspond to the invasion of malignant cells into the subcapsular sinus via lymphatic vessels (LV), as well as a lack of blood supply which leads to necrosis of the medulla [12, 19–21]. Spotted enhancement was linked to dilated subcapsular sinuses whereas stripped pattern is considered an indicator of interlinked capillary sinus. Core and homogenous enhancements are strongly associated with negative LNs, being known as benign patterns [12].

The aim of this study was to perform an in-house validation of the map of Yamamoto et al., which reliability was previously confirmed by the team for synchronous CRCs [9, 16], and to check the correlation between CT-scan-based criteria of suspicion of LN metastases and microscopic features.

## 2. Materials and Methods

### 2.1. Criteria of Inclusion

This prospective study included 112 consecutive patients with CRC, diagnosed and surgically treated by the same surgical team, at the Emergency Clinical County Hospital of Targu Mures, Romania, between 2016 and 2020. The Approval of the Ethical Committee of the Clinical County Emergency Hospital of Targu-Mures, Romania, was obtained for the study. From each patient, signed informed consent was obtained prior surgery for both permissions to perform surgical resection and use of patient information in the scientific publications.

All patients had previous colonoscopy with a positive biopsy for carcinoma. They were referred to the Imaging Department for CRC staging before surgery. We have included all adult patients with preoperatively proved biopsy of carcinoma, in which colectomy and tumor excision was done with free proximal and distal resection margins and extensive lymphadenectomy. Criteria of exclusion: patient's refusal, preoperative oncologic therapy, inoperable cases, death before one month after surgery, associated peritoneal carcinomatosis, recurrent carcinomas, synchronous or metachronous cancers, and independently by their localization, same as diagnosis of a metastatic tumor or a rare histological variant (e.g., neuroendocrine or clear cell carcinoma).

### 2.2. Image Acquisition

In all patients, nonenhanced (NECT) and contrast-enhanced CT (CECT) scans, to identify the localization and characteristics of the tumor and the suspicious LNs, were done. Imagistic investigations were performed by the same team (SP, SI), same as the surgical intervention (BT) and histopathological assessment (GS, JI, BL, and SC).

A Siemens Somatom 64 channel CT scanner was used for the acquisition of images. An abdominopelvic multiphasic CT scan was performed for each patient, with nonenhanced sequence exam followed by intravenous contrast media administration and another two acquisitions: a late arterial phase, at 25 seconds after injection, and a portal-venous phase, at 70 seconds after injection of the contrasting substance. The kilovoltage ranged between 120 and 140 kV with 220 mAs. All patients have received iodinated hydrosoluble contrast media (Optiray 350, 350 mg I/ml) in a dose of 1 ml/kg of body weight with a flow rate ranging from 2 to 3 ml/sec.

### 2.3. Imaging Assessment

**Figure 1:**
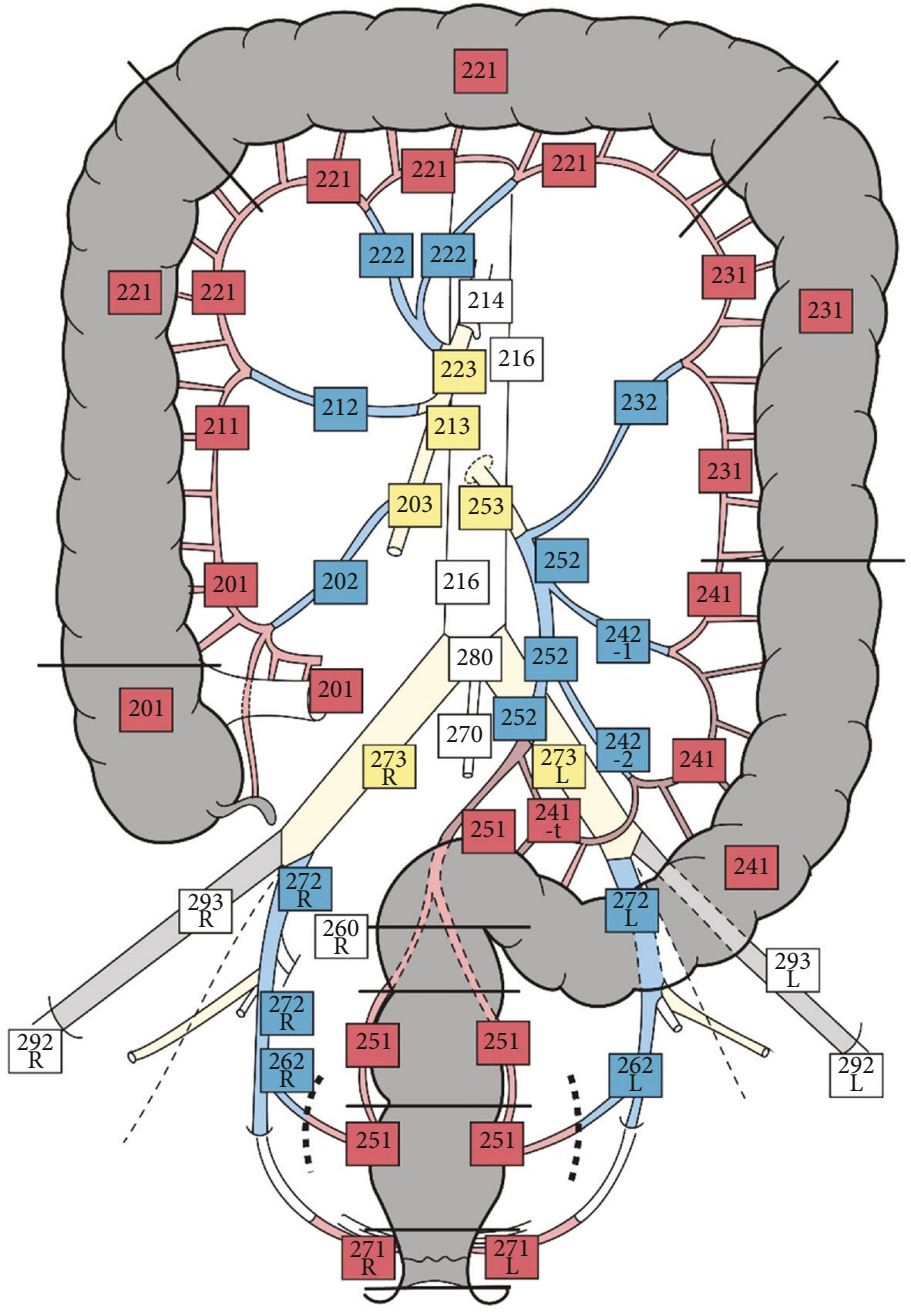
The nodal stations map, adapted with permission from the JSCCR study “Japanese Classification of Colorectal, Appendiceal, and Anal Carcinoma: the 3rd English Edition” [16]. Three categories of nodal stations are seen: locoregional (red), intermediate (blue), and central (yellow and white).

All the abdominopelvic LNs were assessed according to an in-house established protocol, based on the Japanese Classification of Colorectal, Appendiceal, and Anal Carcinoma (JCCRC) developed and updated in 2019 by the Japanese Society of Cancer of the Colon and Rectum (JSCCR). First, a map was adapted from JSCCR study “Japanese Classification of Colorectal, Appendiceal, and Anal Carcinoma: the 3rd English Edition” [16] with permission obtained from the authors, where the nodal stations were divided into three categories: locoregional—within 5 cm from tumor, intermediate-between 5 and 10 cm from tumor, alongside the great vessels, and central LNs—more than 10 cm from tumor, at the origin of great vessels (Figure 1). The map was previously used by our team for identification and evaluation of synchronous CRCs [9]. In the present study, for any patient, an individualized map was done and the LNs suspected of metastases were ringed, to be further harvested by the surgeons.

To consider a LN as being suspected to present metastases, imaging features like short-axis diameter, shape, structure, and borders were considered. The LNs were divided into three groups according to their size: ˂5 mm, 5-10 mm, and ˃10 mm. Suspicion criteria (roundness, heterogeneous density, and irregular border) were considered depending on their size. So, if a LN was ˂5 mm, it needed the presence of all three criteria of suspicion; LNs between 5 and 10 mm were considered suspicious if they had two of three criteria and LNs ˃10 mm were always considered suspicious [12, 18]. The total number of LNs was noted in each case, with the number of suspicious LNs outlined.

**Figure 2:**
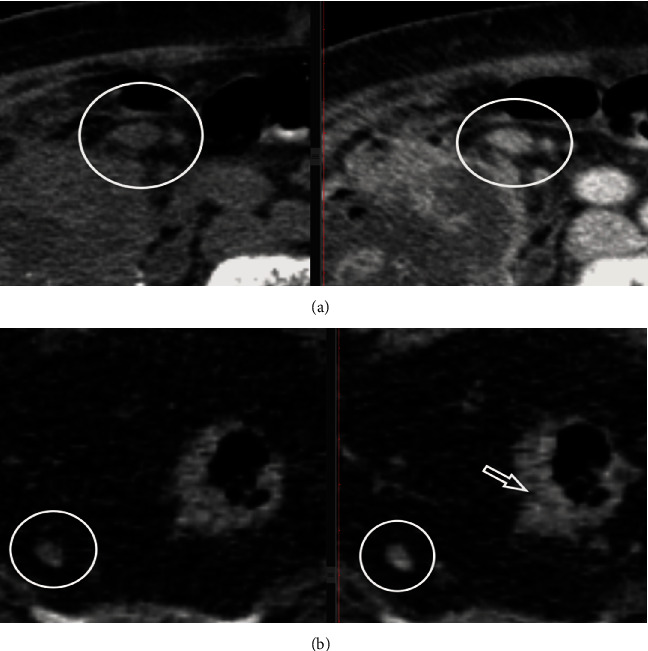
Lymph node assessment on NECT axial scan (left) and on CECT venous phase, axial view (right), with homogenous (a) and dotted enhancement pattern (b); infiltrative tumor of the rectal wall on the right side infiltrates the mesorectal fat (arrow).

**Figure 3:**
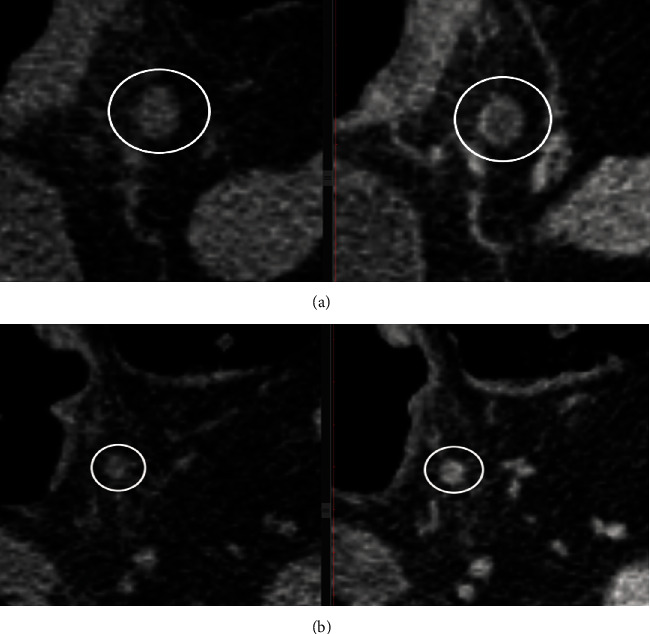
Lymph node assessment on NECT (left) axial view and on CECT scan venous phase, axial view (right), with peripheral (a) and linear enhancement pattern (b).

For an objective evaluation, we combined the previously mentioned features with the enhancement pattern of LNs in the venous phase of CECT, using magnified images. Based on the modified criteria proposed by Miao et al. [12], five patterns of enhancement were checked: homogenous, dotted, linear, central, and peripheral. Dotted pattern was characterized by small spots (<3 mm) of contrast enhancement within the node. Linear pattern was defined as multiple belts of low enhancement giving it a stripey appearance. Central pattern appeared as bright spot of contrast enhancement in the central region, and peripheral pattern was defined as a hypodense center with a hyperdense rim (Figures 2 and 3). The enhancement pattern of both suspicious and nonsuspicious LNs was noted to be then correlated with the pathological reports.

### 2.4. Surgical Interventions and Histopathological Assessment

In all patients, open laparotomy was done for colectomy and surgical removal of the tumor with free proximal and distal resection margins. Based on the imaging map, the LNs which were encircled by the radiologists were harvested in individual recipients, on stations, and send for histopathological assessment [9]. In cases where the encircled nodal stations were peritumoral, the pathologist was informed to check the nodes in the resected surgical specimen.

Gross findings of the surgical specimens were done according to the current guidelines and imaging map. After formalin fixation, the palpable LNs were included for histological examination and comparison of imaging and microscopic features. The encircled suspicious LNs were included in individual histological cassettes. Then, histological slides from formalin-fixed paraffin-embedded tissue (FFPE) blocks were used for current histological assessment. When necessary, immunohistochemical stains with cytokeratin AE1/AE2 were performed for identification of occult metastases or micrometastases.

The pathological reports included the number of LNs harvested according to the nodal stations map and divided into the three groups (˂5 cm, between 5 and 10 cm, and ˃10 cm from tumor), as well as the number of positive LNs. Histological types, pTNM stage, number of LN metastases per node stations, and LNR were also mentioned in the histopathological report. The pTNM stage was established according to the American Joint Committee on Cancer staging system-8^th^ edition (AJCC). Distant node metastases were considered as distant metastases (pM1). Dimensions of the tumors (length and thickness), presence of vascular (V1), lymphatic (L1), and perineural invasion (n1) invasion same as the quality of the resection margins and the tumor budding degree (b) [22] were also pointed for further statistical processing.

### 2.5. Statistical Evaluation

The imaging assessment of the three categories of nodal stations was compared with the pathological reports and divided into four groups: positive correlation (preoperative suspicious nodes were histopathological proved metastatic), negative correlation (no suspicious nodes on imaging and no positive LNs after microscopic evaluation), false positive correlation (suspicious LNs on CT were not found positive on histology report), and false negative correlation (positive LNs under microscope were not matched by suspicious criteria on imaging assessment) [9]. The cases were then categorized based on the suspicious-to-positive ratio (SPR), which was the ratio between the number of CT-suspicious LNs and histologically positive LNs. SPR was calculated for each of the three groups of LNs (˂5 cm, between 5 and 10 cm, and ˃10 cm from tumor). Demographic (age, gender), imaging (suspicious and non-suspicious LNs), and histopathological parameters were compared between cases with positive vs. negative LNs. Sensitivity and specificity were calculated, as well as positive and negative predicted values (PPV, NPV). Statistically significant differences were considered for p˂0.05.

## 3. Results

### 3.1. Clinicopathological and Histological Aspects

From 112 patients with CRC, there were 78 males and 34 females (M : F ratio = 2.29 : 1), with a mean age of 65.60 ± 10.99 years (range 27-88 years). Most of the patients (71.42%) were diagnosed below their 60. LN metastases (LNM) were identified in one-third of the cases. Distant metastases were also seen in over one-quarter of the patients (Table 1). Mean length of the tumors was 48.14 ± 20.61 cm, whereas tumor thickness was of 20.07 ± 11.85 cm. There were 61 patients with rectal- and 51 with colon cancer. Risk of LNMs was not associated with the tumor localizations but was higher in pV1L1n1b3 cases (Table 2).

### 3.2. Preoperative Imaging Assessment of LNs

**Figure 4:**
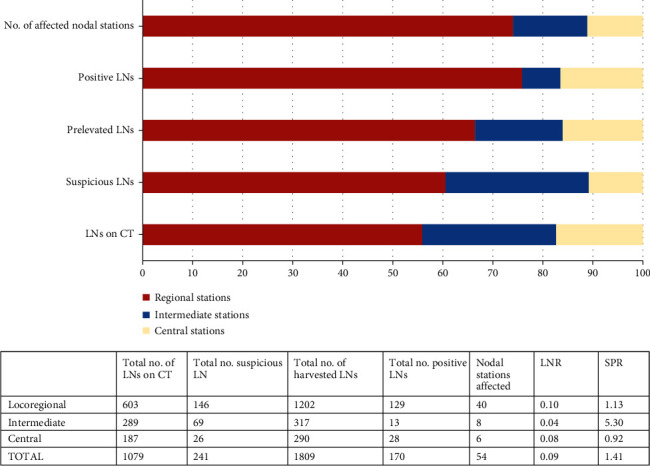
Distribution of LNs, identified on CT scan, per stations, based on the imaging map, and their correlation with the histopathological findings (LNR: lymph node ratio; SPR: suspicious-to-positive ratio).

Based on the CT-scan assessment, 1079 LNs were identified. Most LNs were seen in the first category of nodal stations—locoregional (n = 603; 33.33%), which showed a LNR of 0.10. From the 1079 nodes, 241 (22.34%) were considered “suspect of metastases,” according to the imaging protocol and were encircled on the nodal stations map. Most suspicious LNs were locoregional (n = 146; 60.58%) (Figure 4). The commonest suspicion criteria were roundness (64.56%), followed by inhomogeneity (22.75%) and irregular borders (12.67%).

**Figure 5:**
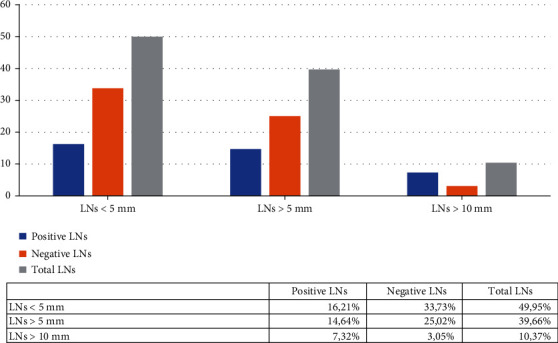
Distribution of metastatic and non-metastatic LNs based on their size.

The median size of the LNs was 6 ± 2.34 mm (range 2-34 mm). Almost half of them were ˂5 mm. LNs˂10 mm were more likely to be negative (Figure 5).

### 3.3. Intra- and Postoperative Assessment of LNs

From the 112 cases, a total of 1809 LNs were removed during surgery and examined under microscope. The median value of the harvested nodes was 15 ± 1.34 LNs/case (range between 1 and 60 nodes); over 14 nodes were successfully retrieved in 75 cases (66.96%). From the 1809 nodes, 170 were metastatic (9.39%) (Figure 4). A percentage of 65.31% of LNs˂10 mm was nonmetastatic but 70.53% of those exceeding 10 mm showed metastases at microscopic examination (Figure 5).

The NPV was 0.92 overall, with a sensitivity and specificity of 80% and 69%, respectively. The PPV was lower, being calculated at 0.42 (Table 3).

**Figure 6:**
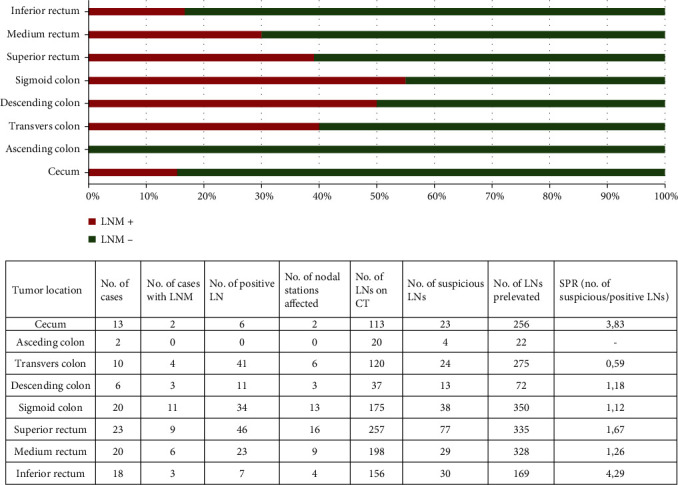
Distribution of lymph nodes (LNs) based on the location of the primary tumor, the number of prelevated/harvested LNs, and suspicious-to-positive ratio (SPR) value.

LNMs were identified in 38 of the 112 patients (33.93%). Majority of the metastatic cases involved sigma (n = 11; metastases predominantly in the station 241) and superior rectum (n = 9; nodal station 251). In most of the cases, positive LNs were seen in one nodal station, respectively, in the first category—locoregional (n = 29; 74.35%). There were 6 cases with two positive nodal stations, 3 cases with three and one case with four nodal stations with LNMs. The SPR value was 1.13 for locoregional, 5.30 for intermediate, and 0.92 for central node stations. The most accurate SPR values were obtained for the cases located on the sigmoid and descending colon same as for those of the middle rectum (1.12; 1.18; 1.26) (Figure 6).

### 3.4. Internal Enhancement Pattern Analysis

Both NECT and CECT examinations were used for a more objective CT-histology correlation. The homogenous (43.72%) and linear (28.83%) enhancement patterns were predominant, being more likely met in negative LNs (76.38% vs. 23.61% for homogenous pattern and 72% vs. 28% for linear pattern). Dotted, peripheral, and central patterns were rather encountered in metastatic LNs (Table 4).

### 3.5. Follow-Up and Survival

Follow-up of the patients was made for 21.61 ± 10.61 months. From the total of 112 patients, 79 (70.53%) survived over 20 months. No gender predilection was observed. Looking at the age distribution, a statistical difference was seen between patients younger or older than 60 years (p < 0.05, p: 0.024, CI 95%). At 20 months after surgery, the highest OS was seen for stages I (75%) and II (86.66%), followed by stages III (64%) and IV (37.5%). LN status proved to have independent prognostic value.

## 4. Discussion

This study confirmed the fact that a transdisciplinary approach of CRC diagnosis and therapeutic management can successfully improve the staging accuracy. It also confirmed the independent prognostic value of LN status and the role of vascular, lymphatic, and perineural invasion, and same as tumor budding degree for predicting the risk of LNMs [22–25]. Although the diagnostic techniques have been improved and certain pathological and molecular markers have been found to have an impact on prognosis, the 5-year OS does not exceed 68% for patients with LNMs [10]. Since the main therapeutic approach remains surgery, one of the most crucial points in staging of CRC is preoperatively identification of the suspicious LNs and removal of the suspect nodes.

In our study, the preoperative imaging assessment was done according to the JCCRC, developed and updated in 2019 by JSCCR guidelines [16]. Each case was evaluated by a team of radiologists, surgeons, and pathologists, with an in-house protocol and a map of LN stations that helped making the correlations between imaging and pathological features.

An important and recognized independent prognostic factor, for patients with CRC, is also the number of harvested LNs [7, 26]. At least 12 LNs are indicated to be evaluated but LNR needs to be also counted [7, 27]. The method proposed in this study was successfully proved to enhance the number of identified LNs per case till 15, with a LNR of 0.10 for regional nodes.

On the other hand, although several studies showed that size of the LNs is not an adequate parameter to predict nodal involvement [18, 26], a cut-off value of 10 mm showed a sensibility and specificity of 71% and 67%, respectively [13]. Size alone fails to be an accurate predictor for node metastasis. It can be an indicator of suspicion only if it is combined with the other parameters such inhomogeneity, irregular borders, heterogeneous density same as presence of dotted, peripheral, or central enhancement pattern. We noticed that, since almost half of the LNs were under 5 mms, the size makes difficult distinguishing spots of enhancement under or over 3 mm. Comparing our findings with those previously reported by Miao et al. [12], our dotted pattern was similar to the previously called spotted pattern, it being correlated with positive LNs (OR: 7.84 and p < 0.000100). Homogenous pattern was associated with nonmetastastic LNs (OR: 1.99, p < 0.05, p: 0.02).

In this cohort, the sensitivity and specificity of CT-scan evaluation were 80% and 69%, respectively. A similar sensitivity but a better specificity (97%) was previously reported [28, 29]. It can be explained but the fact that MRI was used by Brown et al., which has a greater accuracy in depicting differences of signal in nodes [29]. Despite using a more convenient method, our NPV was quite high (0.92), meaning that nonsuspicious LNs were more likely to be negative.

The SPR is a parameter which was introduced by the authors' team to evaluate the correlation between suspicious LNs on CT-scan and positive LNs confirmed under microscope. We noticed the most accurate SPR values for tumors located in the sigmoid colon, descending colon, and middle rectum (1.12, 1.18, and 1.26, respectively). For these cases, the majority of LNs with suspicious criteria on CT were confirmed as positive by pathologist, notwithstanding the fact that the number of cases in the descending colon category was significantly lower than the other two groups. At the opposite pole, tumors localized at the level of transverse colon had SPR value below 1 (0.59), meaning that CT failed to identify all the positive LNs, using only the classic criteria of suspicion. It highlights the limitations of CT scan in some cases. This observation was first time highlighted in literature.

There were some limitations of the study. For more statistically significant results, further research for longer periods on larger cohorts of patients using a standardized preoperative evaluation protocol is necessary. It should include CT acquisition and examination of nodal stations based on the enhancing pattern. MRI confirmation of the data would increase the significance of the proposed method. All the imaging data must be correlated with histological reports to also highlight the morphological changes which might interfere with the enhancement pattern.

## 5. Conclusions

In-house validation of the mapping of the nodal stations affected by CRC might be an important tool of raising the accuracy of detecting the number of suspected LNs. Assessment of the SPR values could be a key in prognosis of these patients, especially for those with tumors of the distal colon and middle rectum.

## Figures and Tables

**Table 1:** Clinicopathological parameters of the examined colorectal cancers (G-grade of differentiation).

Variable	Number (n = 112)	Percentage (%)
Histological type (i) Adenocarcinoma-G1 (ii) Adenocarcinoma-G2 (iii) Adenocarcinoma-G3 (iv) Mucinous carcinoma	(i) 3 (ii) 54 (iii) 5 (iv) 40	(i) 2.68 (ii) 48.21 (iii) 4.47 (iv) 44.64
Depth of infiltration (T stage) (i) T1 (ii) T2 (iii) T3 (iv) T4	(i) 4 (ii) 13 (iii) 57 (iv) 38	(i) 3.57 (ii) 11.61 (iii) 50.89 (iv) 33.93
Lymph node status (N stage) (i) N0 (ii) N1 (iii) N2	(i) 73 (ii) 23 (iii) 16	(i) 65.18 (ii) 20.54 (iii) 14.29
Distant metastases (M stage) (i) M0 (ii) M1	(i) 94 (ii) 18	(i) 83.93 (ii) 16.07
AJCC staging (TNM) (i) I (ii) II (iii) III (iv) IV	(i) 12 (ii) 45 (iii) 39 (iv) 16	(i) 10.71 (ii) 40.18 (iii) 34.82 (iv) 14.29

**Table 2:** Distribution of lymph node metastasis (LNM) upon clinicopathological parameters.

Variable	LNM + (N1 + 2)	LNM–(N0)	p value
Gender Male (n = 78) Female (n = 34)	(i) 29 (ii) 10	(i) 49 (ii) 24	0.52
Tumor localization (i) Cecum (n = 13) (ii) Ascending (n = 2) (iii) Transverse (n = 10) (iv) Descending (n = 6) (v) Sigmoid (n = 20) (vi) Superior rectum (n = 23) (vii) Middle rectum (n = 20) (viii) Inferior rectum (n = 18)	(i) 2 (ii) 0 (iii) 4 (iv) 3 (v) 11 (vi) 9 (vii) 6 (viii) 3	(i) 11 (ii) 2 (iii) 6 (iv) 3 (v) 9 (vi) 14 (vii) 14 (viii) 15	0.15
Vascular invasion (V) (i) V1 (n = 29) (ii) V0 (n = 83)	(i) 16 (ii) 23	(i) 13 (ii) 60	0.01
Lymphatic invasion (L) (i) L1 (n = 46) (ii) L0 (n = 66)	(i) 32 (ii) 7	(i) 14 (ii) 59	˂0.0001
Perineural invasion (n) (i) n1 (n = 25) (ii) n0 (n = 87)	(i) 18 (ii) 21	(i) 7 (ii) 66	˂0.0001
Budding degree (b) (i) b2 + 3–≥5 buds/20 HPF (n = 67) (ii) b1 − ≤5 buds/20 HPF (n = 45)	(i) 35 (ii) 14	(i) 32 (ii) 31	0.03

**Table 3:** Chi-square test showing association between CT-scan suspected node rate and histologic examination (LNM: lymph node metastases).

	LNM +	LNM -	Marginal row totals
Suspicious LNs	156 (92.02) [44.48]	85 (148.98) [27.47]	241
Nonsuspicious LNs	256 (319.98) [12.79]	582 (518.02) [7.9]	838
Marginal column totals	412	667	1079 (grand total)
The chi-square statistic is 92.6485. The p value is < 0.00001. Significant at p < .05.			

**Table 4:** Distribution of enhancement patterns in metastatic vs. non-metastatic nodes (OR: odds ratio, CI 95%).

Enhancement pattern	OR	Lower	Upper	p value
Homogenous	1.99	1.09	3.62	0.02
Dotted	7.84	3.17	19.38	<0.0001
Linear	0.48	0.20	1.14	0.09
Central	2.85	0.40	20.14	0.29
Peripheral	3.25	0.23	44.69	0.37

## Data Availability

The clinicopathological data used to support the findings of this study are available from the corresponding author upon request.
